# Sex-specific association between lumbar spine-total hip T-score discordance and all-cause mortality: a prospective cohort study from NHANES

**DOI:** 10.1007/s11657-026-01729-2

**Published:** 2026-06-23

**Authors:** Rıdvan Erten, Mehmet Poyrazer

**Affiliations:** 1 Division of Geriatrics, Department of Internal Medicine, Faculty of Medicine, Kocaeli University, Kocaeli, 41380 Turkey; 2 Division of Endocrinology and Metabolism, University of Health Sciences Ankara Training and Research Hospital, Ankara, Turkey

**Keywords:** Bone mineral density, Total hip, T-score discordance, Mortality, NHANES, DXA, Sex differences

## Abstract

**Summary:**

In 6364 US adults aged ≥ 50 with up to 15 years of follow-up, lumbar spine–total hip T-score discordance was associated with all-cause mortality in women but not in men. Total hip T-score was the strongest single-site predictor in both sexes.

**Purpose:**

Lumbar spine bone mineral density may be influenced by degenerative and non-skeletal mineralization in older adults. Whether the discrepancy between lumbar spine and hip T-scores carries prognostic information for mortality remains unclear. We examined the sex-specific association of the lumbar spine–total hip T-score difference (ΔT_LS − TH) with all-cause mortality, alongside site-specific T-scores at the lumbar spine, femoral neck, and total hip.

**Methods:**

Prospective cohort study of 6364 adults aged ≥ 50 years from five NHANES cycles (2005–2018) linked to the National Death Index through December 31, 2019. Survey-weighted Cox models estimated hazard ratios (HR) for ΔT (LS T-score minus TH T-score), adjusting for age, sex, BMI, eGFR, smoking, and albumin. Sex-specific associations and a sex × ΔT interaction were tested. Candidate joint models were compared to evaluate whether ΔT_LS − TH provided information beyond total hip T-score. Sensitivity analyses included landmark exclusion of early deaths and additional adjustment for baseline comorbidities**.**

**Results:**

Over a median follow-up of 9.3 years, 1155 deaths occurred. A significant sex × ΔT_LS − TH interaction was observed (*p* = 0.001). In women, ΔT_LS − TH was associated with higher all-cause mortality (HR 1.259 per unit; 95% CI 1.146–1.382; *p* < 0.001), and comparative models favored ΔT_LS − TH over the conventional lumbar spine–femoral neck difference. In men, the corresponding estimate was smaller and did not meet the conventional threshold for statistical significance (HR 1.084 per unit; 95% CI 0.9998–1.175; exact *p* = 0.0504). Total hip T-score was the strongest single-site predictor in both sexes (women HR 0.751, *p* < 0.001; men HR 0.854, *p* = 0.002). Lumbar spine T-score showed no association with mortality in women after multivariable adjustment. Parallel analyses using raw BMD values supported the same site-specific ranking, with total hip BMD showing the strongest single-site association.

**Conclusion:**

In older US adults, the lumbar spine–total hip T-score difference was associated with all-cause mortality in women but not in men, while total hip T-score remained the strongest single-site predictor in both sexes. These findings are consistent with the clinical emphasis on hip measurements when interpreting DXA results in older adults. ΔT_LS − TH may identify a higher-risk pattern of site-specific discordance at the population level, but it should not be used for individual mortality prediction.

**Supplementary Information:**

The online version contains supplementary material available at 10.1007/s11657-026-01729-2.

## Introduction

Dual-energy X-ray absorptiometry (DXA) of the lumbar spine (LS) and femoral neck (FN) is the cornerstone of osteoporosis diagnosis and fracture risk assessment [1]. In clinical practice, however, T-scores at these two sites frequently disagree. Discordance between sites categorized into different diagnostic categories at the LS and femoral neck (FN) or total hip (TH) has been described in 30–50% of subjects undergoing DXA [2]. This discordance is not simply a measurement nuisance; it has tangible clinical consequences, complicating treatment decisions and risk stratification [2].

Lumbar spine measurements in older adults may be affected by degenerative change and other non-skeletal influences, including vertebral osteophytes, facet joint sclerosis, and aortic calcification, which can artifactually elevate apparent BMD without reflecting true skeletal strength [3, 4]. These artifactual elevations have been recognized as a longstanding limitation of LS DXA in older adults [3, 4]. Densitometric practice and DXA interpretation guidelines accordingly emphasize careful clinical interpretation, including monitoring and cross-calibration considerations [5]. Total hip provides an integrated proximal femur measurement that includes both trabecular and cortical compartments and is widely used alongside femoral neck for clinical DXA interpretation in older adults [5].

The difference between LS and hip T-scores can be positive (LS T-score higher than the hip T-score), negative (LS T-score lower than the hip T-score), or neutral when the two sites yield concordant values within a small range. Several investigations have demonstrated that this difference, in its conventional LS − FN form, carries prognostic information for fracture risk. Leslie et al., using data from Manitoba and the Canadian Multicentre Osteoporosis Study (CaMos), showed that the LS–FN T-score offset independently predicted major osteoporotic fractures beyond FRAX probability, with each unit increase in offset associated with a 12–15% increase in fracture risk [6, 7]. Alarkawi et al. reported that women with lower LS BMD relative to FN BMD—that is, negative ΔT, reflecting a discordance pattern opposite to the one examined in the present study—had a 30% higher fracture incidence per standard deviation below FN BMD [8]. In a Korean hip fracture cohort, discordance—particularly with relatively low FN—was significantly more prevalent in fracture patients than in matched controls [9]. These studies established discordance as a fracture-relevant clinical signal but were uniformly framed around the LS–FN axis and around fracture, not mortality, outcomes.

Beyond fractures, low BMD at individual skeletal sites has been linked to all-cause and cardiovascular mortality in population-based studies, including analyses from the National Health and Nutrition Examination Survey (NHANES) [10, 11]. Although spine–hip T-score discordance has been studied primarily in relation to fracture risk, whether a total hip–anchored measure of lumbar–hip discordance carries prognostic information for mortality has not been characterized in a population-based cohort. Because TH integrates a broader proximal femur region than FN, an LS–TH formulation may offer a complementary way to quantify lumbar–hip divergence in older adults.

We therefore aimed (1) to describe the distribution of lumbar spine–total hip T-score discordance in US adults aged ≥ 50 years; (2) to examine its sex-specific association with all-cause mortality; and (3) to compare its prognostic information with site-specific lumbar spine, femoral neck, and total hip T-scores, including femoral neck–anchored discordance models for consistency with prior literature.

## Methods

### Study population and design

This prospective cohort study used data from five cycles of the continuous NHANES: 2005–2006, 2007–2008, 2009–2010, 2013–2014, and 2017–2018. The 2011–2012 and 2015–2016 cycles were excluded because dedicated hip and lumbar spine DXA were not performed in those cycles (only total body DXA was acquired) [12, 14]. NHANES is a nationally representative, cross-sectional health examination survey of the civilian, noninstitutionalized US population, conducted by the National Center for Health Statistics (NCHS) using a complex, stratified, multistage probability sampling design [13].

Eligible participants were adults aged 50 years and older with valid BMD measurements at the lumbar spine (L1–L4), femoral neck, and total hip, who were eligible for mortality follow-up through the NDI, and who had a follow-up time greater than zero. Participants with missing data on core covariates (body mass index, estimated glomerular filtration rate, serum albumin, and smoking status) were excluded from the primary analysis. The final analytic sample comprised 6364 participants.

### DXA measurements and T-score computation

DXA scans of the LS (L1–L4) and proximal femur were acquired in NHANES mobile examination centers using Hologic QDR 4500 A fan-beam densitometers (2005–2010) and Hologic Discovery A densitometers (2013–2018; Hologic Inc., Marlborough, MA, USA). Rigorous quality control procedures were maintained, including daily phantom scanning and centralized scan analysis at the University of California, San Francisco [14]. From the proximal femur scan, both femoral neck (FN) and total hip (TH) BMD values were obtained.

T-scores were calculated for each skeletal site using established reference populations of non-Hispanic White women aged 20–29 years. FN T-scores were calculated using the NHANES III young white female reference (age 20–29 years: mean BMD = 0.858 g/cm^2^, SD = 0.120 g/cm^2^) as recommended by the WHO and ISCD [1, 15]. TH T-scores were calculated using the Looker et al. NHANES III proximal femur reference (mean BMD = 0.942 g/cm^2^, SD = 0.122 g/cm^2^) [16]. LS T-scores used the Hologic manufacturer reference for white females (mean BMD = 1.047 g/cm^2^, SD = 0.110 g/cm^2^). The primary exposure was ΔT_LS − TH, defined as the lumbar spine T-score minus the total hip T-score, with positive values indicating relatively higher LS than TH T-scores. The conventional LS–FN difference (ΔT_LS − FN) was retained only as a comparator for consistency with prior discordance literature.

### Mortality ascertainment

Mortality status was ascertained through linkage with the NDI using the NCHS 2019 Public-Use Linked Mortality Files, which provide follow-up through December 31, 2019 [17]. The primary outcome was all-cause mortality. Cause-specific mortality was classified using the underlying cause of death recode (UCOD_LEADING), comprising ten categories: diseases of the heart (code 001), malignant neoplasms (002), chronic lower respiratory diseases (003), accidents (004), cerebrovascular diseases (005), Alzheimer’s disease (006), diabetes mellitus (007), influenza and pneumonia (008), nephritis/nephrotic syndrome (009), and all other causes (010) [17]. Cause-specific Cox models were fitted by treating deaths from causes other than the cause of interest as censored at the time of death. Person-time was calculated from the MEC examination date to date of death or December 31, 2019, whichever occurred first.

### Covariates

Covariates were selected a priori based on known determinants of both BMD and mortality. Age (continuous, years), sex (male/female), and race/ethnicity (non-Hispanic White, non-Hispanic Black, Mexican American, other) were obtained from demographic interviews. Body mass index (BMI, kg/m^2^) and waist circumference (cm) were measured during the physical examination. Smoking status was categorized as ever smoker (≥ 100 lifetime cigarettes) versus never. Serum creatinine and albumin were measured from blood specimens collected during the MEC visit. Estimated glomerular filtration rate (eGFR) was computed using the 2021 CKD-EPI equation without race adjustment [18]. Race/ethnicity and waist circumference were collected for descriptive characterization but were not included in the primary Cox models, which were specified a priori to maintain a parsimonious clinical covariate set. Sensitivity analyses additionally adjusting for race/ethnicity are presented in Online Resource 3.

### Statistical analysis

All primary analyses were conducted using survey-weighted methods to account for the complex sampling design of NHANES. Strata (SDMVSTRA), primary sampling units (SDMVPSU), and MEC examination weights (WTMEC2YR) were specified in the survey design. For pooled analyses across five two-year cycles, weights were divided by five (WTMEC2YR/5), as recommended by NCHS analytic guidelines [19]. Analyses with strata containing a single PSU were handled using the centered adjustment method [20].

Cox proportional hazards models were fitted using the survey-weighted estimator implemented in R (survey::svycoxph) [21]. Hierarchical models were constructed sequentially: Model 1 (unadjusted), Model 2 (age, sex), Model 3 (+ BMI), Model 4 (+ eGFR), and Model 5 (age, sex, BMI, eGFR, smoking, and albumin). In sex-stratified analyses, the same covariate set was used except for sex. Hazard ratios (HRs) and 95% confidence intervals (CIs) were reported per one-unit increase in the exposure. Sex-stratified analyses were performed for women and men separately.

Single-site Cox models were fitted for each of the three site-specific exposures (LS, FN, and TH T-scores) and for the two discordance measures, ΔT_LS − TH (primary) and ΔT_LS − FN (comparator). The primary association of interest in the revised analysis was that of ΔT_LS − TH with all-cause mortality, and a multiplicative sex × ΔT_LS − TH interaction term was tested in the full model. To examine whether ΔT_LS − TH provided incremental information beyond TH alone, joint models including TH T-score and ΔT_LS − TH were fitted. Because ΔT_LS − TH is mathematically derived as the difference of LS and TH T-scores, the joint TH + ΔT_LS − TH model is a reparameterization of the joint TH + LS model: under this reparameterization the LS coefficient equals the ΔT_LS − TH coefficient, while the TH coefficient transforms as β_TH(reparam) = β_TH − β_ΔT. Accordingly, the ΔT_LS − TH term should be interpreted as the lumbar-spine component of risk conditional on total hip T-score, rather than as an independent biological construct. The significance of the added term was assessed using the survey-adjusted Wald test (regTermTest) [21]. Multicollinearity across all candidate joint specifications was systematically assessed using Pearson correlations and variance inflation factors (Online Resource 3, Section S3.1). The shape of the ΔT_LS − TH–mortality association was further explored using sex-specific quartiles with linear trend testing across quartile rank, presented in Online Resource 1.

Sensitivity analyses included a 2-year landmark analysis excluding deaths occurring within the first two years of follow-up to address potential reverse causation [22]; additional adjustment for major self-reported baseline comorbidities, including cardiovascular disease (any of congestive heart failure, coronary heart disease, angina, or myocardial infarction), stroke, cancer, diabetes, hypertension, and chronic lung disease (chronic bronchitis or emphysema, harmonized across all five cycles); parallel models using raw bone mineral density values (g/cm^2^); and femoral neck–anchored discordance models (ΔT_LS − FN) for comparison with prior literature. The primary multivariable model was kept parsimonious and did not include comorbidities because these conditions may function as confounders, intermediates, or markers of systemic disease burden; therefore, comorbidity adjustment was evaluated as a sensitivity analysis rather than incorporated into the primary model.

All-cause mortality was the pre-specified primary outcome. Cause-specific mortality analyses were exploratory and were not designed for definitive inference regarding individual causes of death; formal multiplicity-adjusted testing was not performed. Event counts were tabulated for all ten public-use NHANES leading cause-of-death categories, and survey-weighted Cox models were fitted only for cause-of-death categories with at least 80 events within each sex stratum to avoid unstable estimates from sparse outcomes. The proportional hazards assumption was assessed using Schoenfeld residual-based tests [23] and time-by-exposure interaction terms (Online Resource 3, Section S3.4). Statistical analyses were performed using R version 4.3.3 (R Foundation for Statistical Computing, Vienna, Austria) with the survey (version 4.4–2) and survival (version 3.5) packages.

### Ethics

All NHANES protocols were approved by the NCHS Research Ethics Review Board, and all participants provided written informed consent. The use of de-identified public-use data for this analysis did not require additional institutional review board approval.

## Results

### Study population

**Fig. 1:**
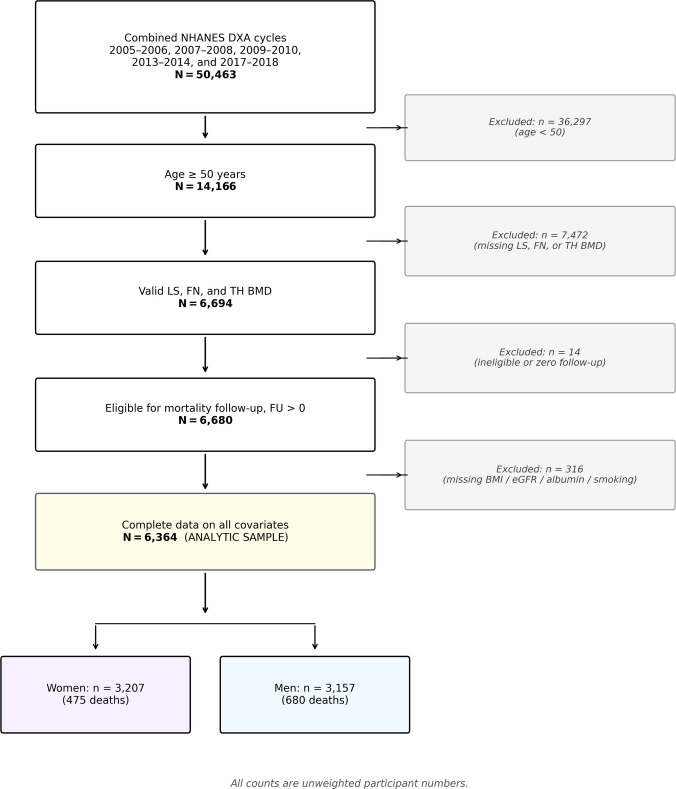
Study flow diagram

Figure 1 shows the participant flow. From 50,463 individuals across five NHANES cycles (2005–2018), 14,166 were aged 50 years or older. Of these, 6694 had valid BMD measurements at the LS, FN, and TH; 6680 were eligible for mortality follow-up with follow-up time greater than zero; and 6364 had complete data on all covariates, constituting the final analytic sample (representing approximately 75.6 million US adults after survey weighting). The weighted proportion of women was 53.3%. The weighted mean age was 61.3 ± 8.9 years and the weighted mean BMI was 28.1 ± 5.5 kg/m^2^. During a median follow-up of 9.3 years (interquartile range 5.1–11.8; total 52,418 person-years), 1155 deaths occurred (weighted mortality proportion 13.9%), including 274 deaths from diseases of the heart and 304 cancer deaths. Survey-weighted baseline characteristics by sex are shown in Online Resource 1, Section S1.1, and characteristics by ΔT_LS − TH quartile in women are shown in Table 1.

**Table 1:** Survey-weighted baseline characteristics of women by ΔT_LS − TH quartile

	Q1 (*n* = 802)	Q2 (*n* = 802)	Q3 (*n* = 801)	Q4 (*n* = 802)
ΔT_LS − TH range	− 3.77 to − 0.95	− 0.95 to − 0.25	− 0.25 to 0.44	0.44 to 4.87
ΔT_LS − TH, mean ± SD	− 1.50 ± 0.41	− 0.58 ± 0.20	0.09 ± 0.20	1.13 ± 0.61
Age, years	61.2 ± 8.3	60.9 ± 8.3	61.0 ± 8.6	63.7 ± 9.9
BMI, kg/m^2^	28.4 ± 6.1	28.0 ± 5.8	27.8 ± 6.1	27.7 ± 6.0
eGFR, mL/min/1.73m^2^	85.3 ± 16.6	85.1 ± 16.3	83.1 ± 17.2	79.0 ± 19.0
Serum albumin, g/dL	4.18 ± 0.27	4.19 ± 0.29	4.18 ± 0.30	4.14 ± 0.32
Ever smoker, %	36.2	39.1	36.2	43.9
NH White, %	62.3	74.3	75.9	81.9
LS BMD, g/cm^2^	0.83 ± 0.13	0.91 ± 0.12	0.96 ± 0.12	1.07 ± 0.14
FN BMD, g/cm^2^	0.74 ± 0.13	0.73 ± 0.12	0.71 ± 0.13	0.71 ± 0.13
TH BMD, g/cm^2^	0.89 ± 0.14	0.86 ± 0.13	0.84 ± 0.13	0.83 ± 0.14
LS T-score	− 1.94 ± 1.18	− 1.23 ± 1.09	− 0.75 ± 1.13	0.18 ± 1.25
FN T-score	− 1.01 ± 1.10	− 1.09 ± 1.01	− 1.20 ± 1.08	− 1.25 ± 1.06
TH T-score	− 0.44 ± 1.19	− 0.65 ± 1.09	− 0.83 ± 1.10	− 0.95 ± 1.13
Deaths, n (weighted %)	67 (8.4)	99 (9.9)	104 (10.6)	205 (20.1)
Person-years	6699	6872	6482	6329
Mortality rate,/1000 py	10.0	14.4	16.0	32.4

Baseline characteristics are survey-weighted means ± SD or percentages. Deaths (*n*), person-years, and mortality rates are unweighted; weighted death proportions are shown in parentheses. ΔT_LS − TH = lumbar spine T-score minus total hip T-score

### Distribution of ΔT_LS − TH

The lumbar spine–total hip T-score difference (ΔT_LS − TH) varied substantially across the cohort. In women, the survey-weighted mean was − 0.13 (SD 1.02), with 44.0% showing positive values (LS T-score higher than TH T-score), 56.0% negative, and approximately 38% within ± 0.5 units of concordance. In men, the mean was − 0.38 (SD 1.04), with 34.0% positive and 66.0% negative. The conventional ΔT_LS − FN distribution differed: in women the mean was + 0.29 (SD 1.05), with 59.9% showing positive values, and in men the mean was + 0.49 (SD 1.14), with 66.2% positive. The two discordance measures were correlated (Pearson *r* = 0.87 in women) but yielded distinct prognostic profiles, as detailed below. Discordance was common rather than exceptional: only approximately 37–38% of participants had ΔT_LS − TH values within ± 0.5 T-score units. Full distributions, including additional concordance thresholds, are presented in Online Resource 1.

### Site-specific T-score associations with mortality

**Fig. 2:**
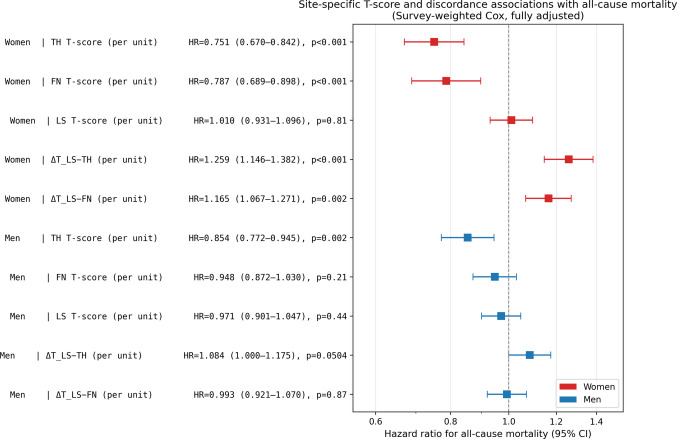
Site-specific T-score and discordance associations with all-cause mortality

Survey-weighted Cox models for each skeletal site are shown in Table 2 and Fig. 2. In women (*n* = 3207; 475 deaths), TH T-score was the strongest single-site predictor of all-cause mortality after full adjustment (HR 0.751 per unit, 95% CI 0.670–0.842; *p* < 0.001), followed by FN T-score (HR 0.787 per unit, 95% CI 0.689–0.898; *p* < 0.001). LS T-score showed no independent association with mortality (HR 1.010 per unit, 95% CI 0.931–1.096; *p* = 0.81). In men (*n* = 3157; 680 deaths), TH T-score was likewise the strongest single-site predictor (HR 0.854 per unit, 95% CI 0.772–0.945; *p* = 0.002), whereas FN T-score (HR 0.948; *p* = 0.21) and LS T-score (HR 0.971; *p* = 0.44) showed no significant association. The TH T-score association differed by sex (sex × TH interaction *p* = 0.017), with a stronger inverse association in women than in men.

**Table 2:** Survey-weighted Cox proportional hazards models for all-cause mortality, by skeletal site and discordance measure

Panel A: Women (*n* = 3207; 475 deaths)			
Exposure	HR per unit	95% CI	*p*
LS T-score	1.010	0.931–1.096	0.81
FN T-score	0.787	0.689–0.898	< 0.001
TH T-score	0.751	0.670–0.842	< 0.001
ΔT_LS − FN (comparator)	1.165	1.067–1.271	0.002
ΔT_LS − TH (primary)	1.259	1.146–1.382	< 0.001
Panel B: Men (*n* = 3157; 680 deaths)			
Exposure	HR per unit	95% CI	*p*
LS T-score	0.971	0.901–1.047	0.44
FN T-score	0.948	0.872–1.030	0.21
TH T-score	0.854	0.772–0.945	0.002
ΔT_LS − FN (comparator)	0.993	0.921–1.070	0.87
ΔT_LS − TH (primary)	1.084	0.9998–1.175	0.0504
Panel C: Sex × exposure interactions			
Interaction term			*p*-value
Sex × ΔT_LS − TH (primary)			0.001
Sex × ΔT_LS − FN (comparator)			0.004
Sex × TH T-score			0.017

All models are survey-weighted (survey::svycoxph) and adjusted for age, body mass index, estimated glomerular filtration rate, smoking, and serum albumin (sex-stratified analyses use the same covariate set without sex). Hazard ratios (HR) are per one-unit increase in the exposure. In men, the ΔT_LS − TH point estimate is smaller in magnitude than in women and the 95% confidence interval included 1.000 (exact *p* = 0.0504); this estimate should not be interpreted as statistically significant. The formal sex-by-exposure interaction (*p* = 0.001) supports effect heterogeneity by sex rather than a uniform association

### ΔT_LS − TH and all-cause mortality

**Fig. 3:**
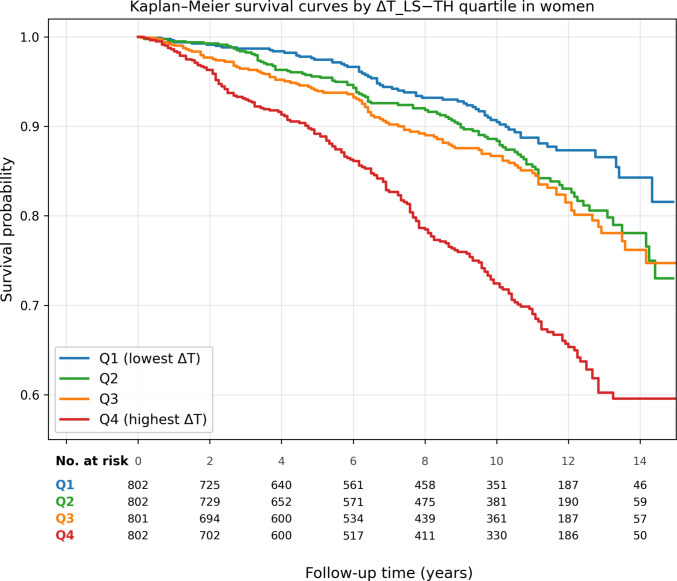
Kaplan–Meier survival curves by ΔT quartile in women. Quartile boundaries: Q1 − 3.77 to − 0.95; Q2 − 0.95 to − 0.25; Q3 − 0.25 to 0.44; Q4 0.44 to 4.87. Numbers at risk are shown below the plot. Curves are unweighted descriptive estimates; primary inference is based on survey-weighted Cox models reported in Table 2

A significant sex × ΔT_LS − TH interaction was observed in the fully adjusted model (interaction *p* = 0.001), indicating a stronger association in women than in men. In women, ΔT_LS − TH was associated with all-cause mortality after multivariable adjustment (HR 1.259 per unit, 95% CI 1.146–1.382; *p* < 0.001). Quartile-based analyses are reported in Online Resource 1. Kaplan–Meier survival curves stratified by ΔT_LS − TH quartile in women are shown in Fig. 3. In men, the corresponding estimate was smaller and borderline but did not meet the conventional threshold for statistical significance (HR 1.084 per unit, 95% CI 0.9998–1.175; exact *p* = 0.0504).

### Joint TH + ΔT_LS − TH model

Table 3 presents the joint model in women including both TH T-score and ΔT_LS − TH. In this specification, TH T-score remained associated with mortality (HR 0.766 per unit, 95% CI 0.682–0.860; *p* < 0.001), and ΔT_LS − TH remained associated with mortality (HR 1.238 per unit, 95% CI 1.123–1.365; *p* < 0.001). The survey-adjusted Wald test confirmed that ΔT_LS − TH contributed statistically detectable incremental information beyond TH alone (*F* = 16.91, *p* < 0.001). Because ΔT_LS − TH is mathematically the difference of LS and TH T-scores, the joint TH + ΔT_LS − TH model is an algebraic reparameterization of the joint TH + LS model: the LS coefficient in the reparameterized model equals the ΔT_LS − TH coefficient (HR 1.238), while the TH coefficient transforms (HR 0.619 in the TH + LS specification). Accordingly, the ΔT_LS − TH term should be interpreted as the lumbar-spine component of risk conditional on total hip T-score rather than as an independent biological construct. Variance inflation factors in the joint TH + ΔT_LS − TH model were low (1.53 and 1.08), indicating no concerning collinearity (Online Resource 3, Section S3.1).

**Table 3:** Joint Cox model in women: total hip T-score and ΔT_LS − TH

Panel A: Joint model (TH + ΔT_LS − TH)			
Exposure	HR per unit	95% CI	*p*
TH T-score	0.766	0.682–0.860	< 0.001
ΔT_LS − TH	1.238	1.123–1.365	< 0.001
Panel B: Incremental information of ΔT_LS − TH beyond TH (survey-adjusted Wald test)			
Test	F	df	*p*
Adding ΔT_LS − TH to base + TH model	16.91	1	< 0.001
Panel C: Equivalent reparameterization as a TH + LS joint model			
Exposure	HR per unit	95% CI	*p*
TH T-score	0.619	0.558–0.686	< 0.001
LS T-score	1.238	1.118–1.371	< 0.001

All models are survey-weighted and adjusted for age, BMI, eGFR, smoking, and serum albumin. The TH + ΔT_LS − TH and TH + LS specifications are algebraically equivalent reparameterizations; under reparameterization the LS coefficient equals the ΔT_LS − TH coefficient, but the TH coefficient changes (β_TH(reparam) = β_TH − β_ΔT). Therefore the ΔT_LS − TH term in Panel A should be interpreted as the lumbar-spine component of risk conditional on total hip T-score, rather than as an independent biological construct. The two specifications span the same linear predictor space; minor differences in reported confidence intervals between Panels A and C reflect re-fitting and rounding of the survey-weighted model output. Variance inflation factors in the joint TH + ΔT_LS − TH model are 1.53 (TH) and 1.08 (ΔT_LS − TH), indicating no concerning collinearity (see Online Resource 3, Section S3.1, for the full multicollinearity assessment)

### Parallel analyses using raw BMD

To address the possibility that T-score derivation introduced systematic bias, parallel models were fitted using raw BMD values (g/cm^2^). In women, the direction, magnitude rank order, and statistical significance of all primary associations were preserved: TH BMD HR 1.41 per 1-SD lower BMD (95% CI 1.23–1.61; *p* < 0.001); FN BMD HR 1.31 per 1-SD lower (95% CI 1.13–1.52; *p* < 0.001); LS BMD HR 0.99 per 1-SD lower (95% CI 0.88–1.11; *p* = 0.81); ΔBMD_LS − TH HR 1.31 per 1-SD higher ΔBMD (95% CI 1.19–1.44; *p* < 0.001). Results in men paralleled the T-score analyses, with TH BMD reaching statistical significance (HR 1.21 per 1-SD lower; 95% CI 1.07–1.37; *p* = 0.002) but FN and LS BMD showing no association. Full raw BMD results are presented in Online Resource 3, Section S3.3.

### Cause-specific mortality analyses

Cause-specific event counts across the ten public-use UCOD_LEADING categories are tabulated in Online Resource 2. In women, three categories met the ≥ 80-event threshold applied within each sex stratum to avoid unstable estimates from sparse outcomes: diseases of the heart (*n* = 106 deaths), malignant neoplasms (*n* = 118), and all other causes (*n* = 128). In these exploratory analyses, higher ΔT_LS − TH was associated with mortality from diseases of the heart (HR 1.363 per unit; 95% CI 1.119–1.662; *p* = 0.002) and all-other-cause mortality (HR 1.275; 95% CI 1.046–1.555; *p* = 0.016), but not with cancer mortality (HR 1.100; 95% CI 0.900–1.345; *p* = 0.35). In men, three categories also met the ≥ 80-event threshold, and none showed a statistically significant association with ΔT_LS − TH (heart disease *n* = 168, HR 1.066, *p* = 0.32; malignant neoplasms *n* = 186, HR 1.069, *p* = 0.31; all other causes *n* = 172, HR 1.094, *p* = 0.27). The remaining seven cause-of-death categories had insufficient events (*n* = 6 to 27 in women; *n* = 8 to 44 in men) for stable multivariable estimation and are reported descriptively only.

### Sensitivity analyses

The primary association between ΔT_LS − TH and mortality in women was robust across an extensive set of sensitivity analyses (Online Resource 3). In the 2-year landmark analysis (excluding 357 women with follow-up < 2 years), ΔT_LS − TH remained associated with mortality (HR 1.254, 95% CI 1.139–1.381; *p* < 0.001), and the joint TH + ΔT_LS − TH model retained both effects. Additional adjustment for major self-reported baseline comorbidities (cardiovascular disease, stroke, cancer, diabetes, hypertension, and chronic lung disease) in 6128 participants with complete comorbidity data yielded a comparable estimate: ΔT_LS − TH HR 1.212 (95% CI 1.102–1.334; *p* < 0.001) compared with 1.248 in the same subsample without comorbidity adjustment. The sex × ΔT_LS − TH interaction remained statistically significant after comorbidity adjustment (*p* = 0.003) and in the landmark analysis (*p* = 0.001). The proportional hazards assumption was satisfied for the primary exposure ΔT_LS − TH; the global test indicated modest model-level deviation, and the Cox estimate was therefore interpreted as an average association over follow-up (Online Resource 3, Section S3.4) [23]. Additional adjustment for race/ethnicity, NHANES cycle, or both did not materially alter the women-specific ΔT_LS − TH association; estimates were also consistent across age-restricted subgroups, including women aged ≥ 60 (HR 1.270, 95% CI 1.151–1.401, *p* < 0.001) and women aged ≥ 65 (HR 1.262, 95% CI 1.142–1.395, *p* < 0.001), supporting the relevance of the association among women aged ≥ 50 years and in older age subgroups. For comparison with prior literature, the conventional ΔT_LS − FN was also associated with mortality in women in the fully adjusted model (HR 1.165 per unit, 95% CI 1.067–1.271; *p* = 0.002); the TH-anchored ΔT_LS − TH showed a larger effect estimate and more favorable joint-model diagnostics than the FN-anchored alternative (Online Resource 3, Section S3.2).

## Discussion

In a nationally representative US cohort of 6364 adults aged ≥ 50 years, we identified a sex-specific mortality signal in lumbar spine–total hip T-score discordance that was absent at any single skeletal site in older women—a finding with implications for how DXA results are interpreted in older women. Specifically, the lumbar spine–total hip T-score difference (ΔT_LS − TH) was associated with all-cause mortality after multivariable adjustment in women but not in men, while total hip T-score was the strongest single-site predictor of mortality in both sexes. The lumbar spine T-score, by contrast, showed no independent association with mortality in older women despite its conventional role in osteoporosis assessment. The ΔT_LS − TH association in women retained statistical significance after sequential adjustment for sociodemographic, anthropometric, renal, behavioral, and nutritional covariates, after additional adjustment for major self-reported baseline comorbidities, and after a 2-year landmark exclusion of early deaths.

Three findings distinguish this work from prior literature. First, although total hip BMD is recognized as a robust skeletal site for clinical decision-making in older adults, the relative prognostic value of LS, FN, and TH in head-to-head comparison within a single nationally representative mortality cohort has not previously been quantified. Second, we evaluated a total hip–anchored parameterization of lumbar–hip T-score discordance (ΔT_LS − TH) and showed that it captures statistically detectable incremental information beyond TH alone (Wald *F* = 16.91; *p* < 0.001), with more favorable joint-model diagnostics than the conventional LS − FN discordance measure used in prior fracture-focused literature [6–9]. Third, we documented a sex-specific pattern in which LS T-score was non-prognostic in women, supporting the well-established clinical view that LS DXA in older adults is influenced by degenerative and non-skeletal factors [3, 4], with mortality-linked epidemiologic data from a nationally representative cohort. We do not interpret these findings as challenging existing densitometric guidance; rather, they extend it by quantifying the prognostic implications of site-specific discordance in older women.

The joint-model results suggest that total hip and lumbar spine components carry directionally distinct mortality information in women. Lower total hip T-score was associated with higher mortality, consistent with hip BMD as a marker of skeletal and systemic vulnerability. In contrast, the lumbar-spine component, when interpreted conditional on total hip T-score, was positively associated with mortality. This pattern is compatible with the possibility that higher apparent lumbar spine T-score in older women may partly reflect non-skeletal mineralization or degenerative change rather than skeletal resilience. Thus, ΔT_LS − TH should not be viewed as an independent biological entity, but as a compact parameterization of two directionally divergent site-specific signals.

The well-recognized confounding of LS DXA in older adults by vertebral osteophytes, facet sclerosis, and aortic calcification offers a plausible explanation for both the null prognostic association of LS T-score and the divergent behavior of LS and TH in joint models [3, 4, 25, 26]. We acknowledge that modern DXA devices partially exclude coarse vascular calcification from the LS region of interest. Residual influence from osteophytes, endplate sclerosis, and smaller calcifications, however, remains a documented source of LS BMD overestimation in older adults [25, 26]. Our findings are compatible with this partial rather than complete confounding, but the cross-sectional measurements available in NHANES cannot directly distinguish skeletal from non-skeletal contributions to LS T-score values. Direct comparison of ΔT_LS − TH with abdominal aortic calcification scores, coronary artery calcium, or radiographic degenerative spine indices would be an important next step.

The exploratory cause-specific analyses showed an association of higher ΔT_LS − TH with mortality from diseases of the heart (HR 1.363; *p* = 0.002) and all-other-cause mortality (HR 1.275; *p* = 0.016) but not with cancer mortality (HR 1.100; *p* = 0.35). This pattern is compatible with, but does not establish, a vascular or systemic aging interpretation of lumbar–hip discordance, since abdominal aortic calcification, coronary artery calcium, and radiographic degenerative spine changes were not directly measured, the all-other-cause category was also associated with ΔT_LS − TH, and the cause-specific analyses were exploratory, event-limited, and not designed for definitive inference regarding individual causes of death.

The total hip is anatomically and biomechanically intermediate between the lumbar spine and femoral neck in that it integrates cortical and trabecular compartments across a broader proximal femur region. In our data, the mean TH T-score was − 0.74 in women and + 0.44 in men. Notably, the mean TH T-score did not lie strictly between LS and FN in either sex: in men, TH (+ 0.44) was numerically higher than both LS (+ 0.06) and FN (− 0.43), and in women TH (− 0.74) was closer to LS (mean − 0.86) than to FN (mean − 1.15). This pattern likely reflects differences in the reference populations used for site-specific T-score calculation rather than a contradiction of the cortical/trabecular composition argument; parallel raw BMD analyses, in which reference effects do not apply, supported the same site-specific ranking with TH as the strongest single-site predictor in both sexes (Online Resource 3, Section S3.3). The principal interpretation that TH is the strongest single-site prognostic measurement is unchanged.

It is important to distinguish our cross-sectional findings from the established literature on post-fracture mortality. Prior studies, including the long-term follow-up of the Dubbo Osteoporosis Epidemiology Study, have consistently reported higher post-fracture mortality in men than in women across both hip and vertebral fracture cohorts [24]. Our study addresses a different question: the relationship between baseline DXA-derived discordance and subsequent all-cause mortality in a general population sample of adults aged ≥ 50, the majority of whom did not experience a fracture during follow-up. The mechanisms underlying these two settings plausibly differ; post-fracture excess mortality in men reflects acute post-event clinical complications and pre-fracture comorbidity burden, whereas the discordance signal in our cohort plausibly reflects the chronic interplay between non-skeletal lumbar mineralization, hip skeletal status, and competing causes of death in older women. The two phenomena should not be conflated.

The clinical implications of these findings should be interpreted cautiously. The effect size of the ΔT_LS − TH association in women, although statistically robust, is modest at the individual level (HR 1.26 per unit). The improvement in model discrimination from adding ΔT_LS − TH to a covariate model containing TH alone was small in absolute terms (Online Resource 3). Our findings should therefore be understood as a population-level epidemiologic signal rather than a basis for individual mortality risk prediction, and they do not support the introduction of ΔT_LS − TH as a clinical risk-prediction tool in current densitometric practice. The findings are compatible with the clinical emphasis on hip measurements when interpreting DXA results in older adults, particularly in older women where the LS T-score alone may carry limited prognostic information.

Importantly, these findings are not an artifact of T-score derivation. Parallel analyses using raw bone mineral density values (g/cm^2^) preserved the direction, magnitude rank order, and statistical significance of all primary inferences. In women, total hip BMD per 1-SD lower was associated with mortality with HR 1.41 (95% CI 1.23–1.61, *p* < 0.001), while lumbar spine BMD showed no independent association (HR 0.99, *p* = 0.81); in men, only total hip BMD reached statistical significance (HR 1.21, *p* = 0.002). The robustness of the principal findings under direct BMD parameterization argues against systematic bias from T-score reference selection or sex-specific reference distributions, and reinforces the interpretation that the observed sex-specific discordance signal reflects a biological rather than methodological pattern.

The strengths of this study include its prospective design with NDI-verified mortality ascertainment, nationally representative sampling, large sample size with over 1100 mortality events and median follow-up exceeding 9 years, comprehensive covariate adjustment, systematic head-to-head comparison of all three skeletal sites and two discordance parameterizations, parallel raw BMD analyses, and an extensive sensitivity analysis battery addressing reverse causation, comorbidity confounding, race/ethnicity, NHANES cycle, and age-group effects.

Several limitations should be acknowledged. *First*, this is an observational study with single-time-point DXA at baseline, precluding causal inference and the assessment of longitudinal BMD trajectories; NHANES does not include prospective fracture data, so we could not assess whether the ΔT–mortality association is mediated by incident fractures. *Second*, the principal interpretive limitation lies with LS DXA itself in older adults: while modern devices partially exclude coarse vascular calcification, residual artifact from osteophytes, endplate sclerosis, and smaller calcifications cannot be excluded, and direct radiographic or computed tomography measures of these confounders were not available. *Third*, cause-specific mortality analyses were exploratory, restricted to categories with sufficient event counts, and were not designed for definitive inference regarding individual causes of death; formal multiplicity-adjusted testing was not performed. Relatedly, all-cause mortality is itself an inclusive endpoint that necessarily captures deaths with no plausible skeletal mechanism, which limits causal interpretation of any single baseline risk factor including ΔT_LS − TH; we retained it as the primary outcome because it is less vulnerable to cause-of-death misclassification than individual UCOD categories and because cause-specific event counts in this analytic sample were too sparse for stable multivariable modelling beyond the three highest-event categories. *Fourth*, residual confounding from unmeasured factors—including physical activity, vitamin D status, frailty, falls history, menopausal status, and medication use such as bisphosphonates and statins—cannot be excluded; we adjusted for major self-reported baseline comorbidities in sensitivity analyses (Online Resource 3, Section S3.5), and the primary associations remained comparable. *Fifth*, several methodological aspects of the data warrant note: the DXA scanner was upgraded between cycles, although adjustment for cycle did not change the associations; reference populations differ across skeletal sites, although these constants do not affect Cox regression coefficients in a proportional hazards framework; the public-use mortality files include perturbed follow-up times for a small number of records to protect confidentiality; and the survey weight specification follows NCHS guidelines but is not a purpose-designed mortality weight. Finally, findings from US adults may not be directly generalizable to other populations.

## Conclusion

In US adults aged ≥ 50 years, the lumbar spine–total hip T-score difference (ΔT_LS − TH) was associated with all-cause mortality after multivariable adjustment in women but not in men, while total hip T-score was the strongest single-site mortality predictor in both sexes. Lumbar spine T-score in older women showed no association with mortality after multivariable adjustment. These findings are compatible with the clinical emphasis on hip measurements when interpreting DXA results in older adults and indicate that the lumbar–hip discordance pattern in older women carries population-level prognostic information not captured by either site alone. ΔT_LS − TH should not be used as an individual mortality risk-prediction tool. External replication in other population cohorts and direct integration with measures of vascular calcification and degenerative spine changes are needed to clarify the underlying mechanisms.

## Supplementary Information

Below is the link to the electronic supplementary material.

ESM 1 (DOCX 43.6 KB)

## Data Availability

All data used in this study are publicly available from the CDC/NCHS website (https://www.cdc.gov/nchs/nhanes/) and the NCHS Data Linkage program (https://www.cdc.gov/nchs/data-linkage/).
