# Supplementary material for: Sex-specific association between lumbar spine-total hip T-score discordance and all-cause mortality: a prospective cohort study from NHANES
Source: Arch Osteoporos. 2026 Jun 23;21(1):95. doi: 10.1007/s11657-026-01729-2 (PMC13290820; doi:10.1007/s11657-026-01729-2)
Supplement: Supplementary file 1 — (DOCX 43.6 KB) [file 11657_2026_1729_MOESM1_ESM.docx]

**Electronic Supplementary Material**

*Sex-Specific Association Between Lumbar Spine–Total Hip T-Score Discordance
and All-Cause Mortality: A Prospective Cohort Study From NHANES*

Rıdvan Erten¹, Mehmet Poyrazer²

¹ Division of Geriatrics, Department of Internal Medicine, Faculty of Medicine, Kocaeli University, Kocaeli, 41380, Turkey
² Division of Endocrinology and Metabolism, University of Health Sciences Ankara Training and Research Hospital, Ankara, Turkey

**Contents**

Online Resource 1. Distribution of ΔT measures and women baseline characteristics by ΔT_LS−TH quartile

S1.1 Survey-weighted baseline characteristics by sex

S1.2 Distribution of ΔT_LS−TH and ΔT_LS−FN in women and men

S1.3 Quartile-based Cox analysis in women with linear trend test

Online Resource 2. Cause-specific mortality

S2.1 Event counts across all ten UCOD_LEADING categories, by sex

S2.2 Survey-weighted Cox models for cause-of-death categories with ≥80 events (women)

S2.3 Survey-weighted Cox models for cause-of-death categories with ≥80 events (men)

Online Resource 3. Methodological supplementary analyses

S3.1 Multicollinearity assessment (correlation matrix and variance inflation factors)

S3.2 Femoral neck–anchored discordance (ΔT_LS−FN) — original parameterization

S3.3 Raw bone mineral density parallel analyses

S3.4 Race/ethnicity, NHANES cycle, age subgroup sensitivity, and proportional hazards

S3.5 Comorbidity-adjusted sensitivity analysis

S3.6 Two-year landmark analysis

**Online Resource 1. Distribution of ΔT measures and women baseline characteristics by ΔT_LS−TH quartile**

**S1.1 Survey-weighted baseline characteristics by sex**

|  | **Women (n=3,207)** | **Men (n=3,157)** |
| --- | --- | --- |
| Age, years | 61.8 ± 8.9 | 60.7 ± 8.8 |
| BMI, kg/m² | 27.9 ± 6.0 | 28.4 ± 4.9 |
| eGFR, mL/min/1.73m² | 83.0 ± 17.5 | 84.6 ± 17.0 |
| Serum albumin, g/dL | 4.18 ± 0.30 | 4.25 ± 0.30 |
| Ever smoker, % | 39.0 | 57.3 |
| LS BMD, g/cm² | 0.95 ± 0.15 | 1.05 ± 0.16 |
| FN BMD, g/cm² | 0.72 ± 0.13 | 0.81 ± 0.13 |
| TH BMD, g/cm² | 0.84 ± 0.14 | 1.00 ± 0.14 |
| LS T-score | −0.86 ± 1.39 | 0.06 ± 1.44 |
| FN T-score | −1.15 ± 1.06 | −0.43 ± 1.10 |
| TH T-score | −0.74 ± 1.13 | 0.44 ± 1.19 |
| ΔT_LS−TH (LS − TH) | −0.13 ± 1.02 | −0.38 ± 1.04 |
| ΔT_LS−FN (LS − FN, comparator) | 0.29 ± 1.05 | 0.49 ± 1.14 |
| Follow-up, years (median) | 9.4 | 9.3 |
| Deaths, n (weighted %) | 475 (12.6) | 680 (15.4) |

*Survey-weighted estimates. ΔT_LS−TH = lumbar spine T-score minus total hip T-score; ΔT_LS−FN = lumbar spine T-score minus femoral neck T-score (retained as a comparator only).*

**S1.2 Distribution of ΔT_LS−TH and ΔT_LS−FN in women and men**

|  | **Mean (SD)** | **% Positive** | **% Negative** | **% Within ±0.5** |
| --- | --- | --- | --- | --- |
| Women, ΔT_LS−TH (primary) | −0.13 (1.02) | 44.0 | 56.0 | ≈38 |
| Men, ΔT_LS−TH (primary) | −0.38 (1.04) | 34.0 | 66.0 | ≈37 |
| Women, ΔT_LS−FN (comparator) | +0.29 (1.05) | 59.9 | 40.1 | ≈30 |
| Men, ΔT_LS−FN (comparator) | +0.49 (1.14) | 66.2 | 33.8 | ≈26 |

*Positive ΔT indicates LS T-score higher than the corresponding hip T-score (TH or FN). Pearson correlation between ΔT_LS−TH and ΔT_LS−FN was r = 0.87 in women. Discordance is common rather than exceptional in this cohort: only approximately 37–38% of participants had ΔT_LS−TH within ±0.5 T-score units.*

**S1.3 Quartile-based Cox analysis in women with linear trend test**

| **Comparison** | **HR (95% CI)** | **p** |
| --- | --- | --- |
| Q2 vs Q1 | 1.228 (0.862–1.749) | 0.26 |
| Q3 vs Q1 | 1.346 (0.966–1.876) | 0.079 |
| Q4 vs Q1 | 1.848 (1.314–2.598) | <0.001 |
| Linear trend (per quartile rank) | 1.225 (1.112–1.350) | <0.001 |

*Survey-weighted Cox models adjusted for age, BMI, eGFR, smoking, and serum albumin. ΔT_LS−TH quartiles defined within women. Linear trend tested by modelling quartile rank as a continuous variable. Quartile boundaries (women): Q1 −3.77 to −0.95; Q2 −0.95 to −0.25; Q3 −0.25 to 0.44; Q4 0.44 to 4.87.*

**Online Resource 2. Cause-specific mortality**

**S2.1 Event counts across all ten UCOD_LEADING categories, by sex**

| **UCOD code** | **Cause of death** | **Women (n)** | **Men (n)** | **Total (n)** |
| --- | --- | --- | --- | --- |
| 001 | Diseases of the heart | 106 | 168 | 274 |
| 002 | Malignant neoplasms | 118 | 186 | 304 |
| 003 | Chronic lower respiratory diseases | 27 | 36 | 63 |
| 004 | Accidents (unintentional injuries) | 6 | 8 | 14 |
| 005 | Cerebrovascular diseases | 27 | 44 | 71 |
| 006 | Alzheimer disease | 26 | 23 | 49 |
| 007 | Diabetes mellitus | 16 | 19 | 35 |
| 008 | Influenza and pneumonia | 12 | 12 | 24 |
| 009 | Nephritis, nephrotic syndrome | 9 | 12 | 21 |
| 010 | All other causes | 128 | 172 | 300 |
|  | Total deaths | 475 | 680 | 1,155 |

*Cause-specific Cox models were fitted by treating deaths from causes other than the cause of interest as censored at the time of death. Models were fitted only for cause-of-death categories with at least 80 events within each sex stratum to avoid unstable estimates from sparse outcomes; the remaining categories were reported descriptively only. These analyses are exploratory and were not designed for definitive cause-specific inference; formal multiplicity-adjusted testing was not performed.*

**S2.2 Cause-specific Cox models for categories with ≥80 events (women)**

| **Cause of death** | **n events** | **HR per unit ΔT_LS−TH** | **95% CI** | **p** |
| --- | --- | --- | --- | --- |
| Diseases of the heart (001) | 106 | 1.363 | 1.119–1.662 | 0.002 |
| Malignant neoplasms (002) | 118 | 1.100 | 0.900–1.345 | 0.35 |
| All other causes (010) | 128 | 1.275 | 1.046–1.555 | 0.016 |

*Survey-weighted Cox models adjusted for age, BMI, eGFR, smoking, and serum albumin.*

**S2.3 Cause-specific Cox models for categories with ≥80 events (men)**

| **Cause of death** | **n events** | **HR per unit ΔT_LS−TH** | **95% CI** | **p** |
| --- | --- | --- | --- | --- |
| Diseases of the heart (001) | 168 | 1.066 | 0.940–1.208 | 0.32 |
| Malignant neoplasms (002) | 186 | 1.069 | 0.941–1.215 | 0.31 |
| All other causes (010) | 172 | 1.094 | 0.933–1.282 | 0.27 |

*Survey-weighted Cox models adjusted for age, BMI, eGFR, smoking, and serum albumin. In contrast to women, no cause-specific category showed a statistically significant association with ΔT_LS−TH in men. The point estimates for all three categories were close to 1.0 and confidence intervals included 1.0, consistent with the borderline overall ΔT_LS−TH association observed in men (HR 1.084, 95% CI 0.9998–1.175, exact p=0.0504; main text Table 2).*

**Online Resource 3. Methodological supplementary analyses**

**S3.1 Multicollinearity assessment (correlation matrix and variance inflation factors)**

**Pearson correlation matrix (women, n=3,207):**

|  | **LS** | **FN** | **TH** | **ΔT_LS−FN** | **ΔT_LS−TH** |
| --- | --- | --- | --- | --- | --- |
| LS T-score | 1.00 | 0.67 | 0.69 | 0.64 | 0.59 |
| FN T-score | 0.67 | 1.00 | 0.89 | −0.14 | −0.09 |
| TH T-score | 0.69 | 0.89 | 1.00 | 0.00 | −0.18 |
| ΔT_LS−FN | 0.64 | −0.14 | 0.00 | 1.00 | 0.87 |
| ΔT_LS−TH | 0.59 | −0.09 | −0.18 | 0.87 | 1.00 |

**Variance inflation factors (VIF) across candidate joint specifications (women):**

| **Joint model specification** | **VIF (predictor 1)** | **VIF (predictor 2)** | **Interpretation** |
| --- | --- | --- | --- |
| FN + ΔT_LS−FN (original parameterization) | 1.45 | 1.06 | Low; clean |
| TH + ΔT_LS−TH (primary parameterization) | 1.53 | 1.08 | Low; lowest among candidates |
| TH + LS (reparameterization of TH+ΔT_LS−TH) | 2.50 | 2.02 | Low |
| TH + FN | 5.35 | 5.06 | Borderline; intra-hip correlation r=0.89 |
| TH + FN + LS | 5.84/5.19/2.08 | — | Borderline |
| ΔT_LS−FN + ΔT_LS−TH | 4.35 | 4.44 | Borderline |

*VIF values calculated from linear regression of each predictor on all other predictors plus the full covariate set within women. The conventional concern threshold is VIF > 5–10.*

**S3.2 Femoral neck–anchored discordance (ΔT_LS−FN) — original parameterization**

For consistency with prior fracture-focused literature, the original LS−FN discordance was retained as a comparator. Survey-weighted Cox results in women (fully adjusted):

| **Model** | **HR per unit** | **95% CI** | **p** |
| --- | --- | --- | --- |
| ΔT_LS−FN alone | 1.165 | 1.067–1.271 | 0.002 |
| FN + ΔT_LS−FN (joint) | — | — | — |
| FN T-score | 0.787 | 0.689–0.898 | <0.001 |
| ΔT_LS−FN | 1.154 | 1.056–1.260 | 0.004 |
| Wald test for added ΔT_LS−FN | F=8.12 | — | 0.006 |

*All models adjusted for age, BMI, eGFR, smoking, and serum albumin. The FN + ΔT_LS−FN parameterization yields a statistically significant association of similar direction and slightly smaller magnitude than the TH-anchored ΔT_LS−TH primary model. Comparison of joint models in women yielded AIC = 6539.7 for FN + ΔT_LS−FN versus AIC = 6514.2 for TH + ΔT_LS−TH (lower is better; same outcome and covariate set, unweighted Cox).*

**S3.3 Raw bone mineral density parallel analyses**

To address whether T-score derivation introduced systematic bias, parallel models were fitted using raw BMD values (g/cm²). For BMD measurements the hazard ratio is reported per 1-SD lower BMD; for the raw BMD discordance ΔBMD it is reported per 1-SD higher ΔBMD.

**Women (n=3,207, 475 deaths)**

| **Exposure** | **HR (per 1-SD)** | **95% CI** | **p** |
| --- | --- | --- | --- |
| LS BMD (per 1-SD lower) | 0.99 | 0.88–1.11 | 0.81 |
| FN BMD (per 1-SD lower) | 1.31 | 1.13–1.52 | <0.001 |
| TH BMD (per 1-SD lower) | 1.41 | 1.23–1.61 | <0.001 |
| ΔBMD_LS−TH (per 1-SD higher) | 1.31 | 1.19–1.44 | <0.001 |
| ΔBMD_LS−FN (per 1-SD higher) | 1.20 | 1.08–1.32 | <0.001 |

**Men (n=3,157, 680 deaths)**

| **Exposure** | **HR (per 1-SD)** | **95% CI** | **p** |
| --- | --- | --- | --- |
| LS BMD (per 1-SD lower) | 1.05 | 0.94–1.17 | 0.44 |
| FN BMD (per 1-SD lower) | 1.06 | 0.96–1.18 | 0.25 |
| TH BMD (per 1-SD lower) | 1.21 | 1.07–1.37 | 0.002 |
| ΔBMD_LS−TH (per 1-SD higher) | 1.12 | 1.02–1.22 | 0.022 |
| ΔBMD_LS−FN (per 1-SD higher) | 0.99 | 0.90–1.11 | 0.93 |

*Survey-weighted Cox models adjusted for age, BMI, eGFR, smoking, and serum albumin. In women, the direction, magnitude ranking, and statistical significance of the primary findings were preserved in raw BMD analyses. In men, the site-specific BMD pattern remained similar, although the raw ΔBMD_LS−TH sensitivity estimate was weakly positive; therefore, the primary sex-specific inference remains based on the pre-specified T-score model. These sensitivity analyses argue against T-score derivation as the sole explanation for the observed pattern.*

**Model discrimination (Harrell’s C-index, women, unweighted Cox):**

| **Model** | **C-index** | **ΔC vs base** | **AIC** |
| --- | --- | --- | --- |
| Base (age + BMI + eGFR + smoking + albumin) | 0.7863 | reference | 6,561.2 |
| + TH alone | 0.7917 | +0.0054 | 6,544.1 |
| + FN alone | 0.7893 | +0.0030 | — |
| + LS alone | 0.7864 | +0.0001 | — |
| + TH + ΔT_LS−TH (primary) | 0.7978 | +0.0114 | 6,514.2 |
| + FN + ΔT_LS−FN (comparator) | 0.7929 | +0.0066 | 6,539.7 |

*Survey-weighted concordance is not directly available; unweighted Cox C-indices reported above are consistent with the survey-weighted Wald test results in the main text. The TH + ΔT_LS−TH primary parameterization shows the lowest AIC and the largest ΔC.*

**S3.4 Race/ethnicity, NHANES cycle, age subgroups, and proportional hazards assumption**

**Sensitivity of the ΔT_LS−TH–mortality association in women:**

| **Sensitivity analysis** | **N** | **Deaths** | **HR (95% CI)** | **p** |
| --- | --- | --- | --- | --- |
| Primary (full adjustment) | 3,207 | 475 | 1.259 (1.146–1.382) | <0.001 |
| + Race/ethnicity | 3,207 | 475 | 1.262 (1.146–1.391) | <0.001 |
| + NHANES cycle | 3,207 | 475 | 1.258 (1.143–1.385) | <0.001 |
| + Race/ethnicity + cycle | 3,207 | 475 | 1.261 (1.144–1.390) | <0.001 |
| Age ≥60 subgroup | 1,975 | 408 | 1.270 (1.151–1.401) | <0.001 |
| Age ≥65 subgroup | 1,260 | 341 | 1.262 (1.142–1.395) | <0.001 |

*All models adjusted for age, BMI, eGFR, smoking, and serum albumin. The ΔT_LS−TH primary association is robust to additional adjustment for race/ethnicity, NHANES cycle, and to restriction by age subgroups.*

**Proportional hazards assumption — Schoenfeld residual-based test (cox.zph applied to unweighted Cox model in women, diagnostic only):**

| **Covariate** | **χ²** | **df** | **p** |
| --- | --- | --- | --- |
| ΔT_LS−TH (primary exposure) | 1.59 | 1 | 0.21 |
| Age | 0.13 | 1 | 0.72 |
| BMI | 2.81 | 1 | 0.094 |
| eGFR | 3.10 | 1 | 0.078 |
| Ever smoker | <0.001 | 1 | 0.98 |
| Albumin | 3.51 | 1 | 0.061 |
| GLOBAL | 13.7 | 6 | 0.034 |

*The proportional hazards assumption was satisfied for the primary exposure ΔT_LS−TH (p=0.21). The global test was borderline (p=0.034), driven primarily by minor non-proportionality contributions from BMI, eGFR, and serum albumin (each individually p>0.05). These diagnostics suggest that the primary exposure did not show material non-proportionality, although the global test indicated modest model-level deviation. Accordingly, the Cox estimate should be interpreted as an average association over follow-up. Sensitivity analyses with shorter follow-up windows (2-year landmark; Section S3.6) yielded ΔT_LS−TH estimates consistent with the primary analysis (HR 1.254, 95% CI 1.139–1.381), supporting the robustness of the primary inference.*

*As an additional diagnostic check, a time-by-exposure interaction term was examined in the survey-weighted Cox model for women. Because this diagnostic analysis did not alter the direction or interpretation of the primary association, the primary Cox estimate was interpreted as an average association over follow-up.*

**S3.5 Comorbidity-adjusted sensitivity analysis**

Self-reported baseline history of cardiovascular disease (any of congestive heart failure, coronary heart disease, angina, or myocardial infarction), stroke, cancer, diabetes, hypertension, and chronic lung disease (chronic bronchitis or emphysema, harmonized across all five cycles) was added to the fully adjusted Cox models. Among 6,128 participants with complete comorbidity data (women n=3,108, 453 deaths; men n=3,020, 652 deaths), point estimates for the primary associations were not materially attenuated.

| **Exposure (women)** | **Original adjustment** | **+ Comorbidity adjustment** |
| --- | --- | --- |
| ΔT_LS−TH (primary) | 1.248 (1.134–1.375), p<0.001 | 1.212 (1.102–1.334), p<0.001 |
| ΔT_LS−FN (comparator) | 1.153 (1.048–1.269), p=0.004 | 1.141 (1.037–1.255), p=0.007 |
| TH T-score | 0.752 (0.668–0.847), p<0.001 | 0.787 (0.699–0.886), p<0.001 |
| FN T-score | 0.792 (0.691–0.907), p<0.001 | 0.819 (0.727–0.923), p=0.001 |
| LS T-score | 1.007 (0.925–1.096), p=0.87 | 1.010 (0.925–1.104), p=0.82 |

**Joint TH + ΔT_LS−TH model with comorbidity adjustment (women, n=3,108, 453 deaths):**

| **Term** | **HR (95% CI)** | **p** |
| --- | --- | --- |
| TH T-score | 0.800 (0.711–0.899) | <0.001 |
| ΔT_LS−TH | 1.196 (1.079–1.326) | <0.001 |

*The sex × ΔT_LS−TH interaction remained statistically significant after comorbidity adjustment (p=0.003). The primary associations were not materially explained by the major self-reported comorbidities measured here.*

**S3.6 Two-year landmark analysis**

To address potential reverse causation arising from undiagnosed disease at baseline, we excluded participants with follow-up shorter than two years and reset the time origin to two years after examination. The landmark sample comprised 5,677 participants.

**Landmark sample sizes:**

| **Group** | **n (landmark)** | **Deaths (landmark)** | **Excluded (n)** | **Excluded deaths** |
| --- | --- | --- | --- | --- |
| Women | 2,850 | 415 | 357 | 60 |
| Men | 2,827 | 580 | 330 | 100 |
| Total | 5,677 | 995 | 687 | 160 |

**Landmark Cox results (women, n=2,850, 415 deaths):**

| **Exposure** | **HR per unit** | **95% CI** | **p** |
| --- | --- | --- | --- |
| ΔT_LS−TH (primary) | 1.254 | 1.139–1.381 | <0.001 |
| ΔT_LS−FN (comparator) | 1.171 | 1.062–1.292 | 0.002 |
| TH T-score | 0.764 | 0.671–0.870 | <0.001 |
| FN T-score | 0.793 | 0.685–0.918 | 0.002 |
| LS T-score | 1.017 | 0.929–1.113 | 0.72 |
| Joint TH + ΔT_LS−TH (TH term) | 0.780 | 0.687–0.885 | <0.001 |
| Joint TH + ΔT_LS−TH (ΔT_LS−TH term) | 1.235 | 1.112–1.372 | <0.001 |

**Landmark Cox results (men, n=2,827, 580 deaths):**

| **Exposure** | **HR per unit** | **95% CI** | **p** |
| --- | --- | --- | --- |
| ΔT_LS−TH | 1.064 | 0.973–1.163 | 0.17 |
| TH T-score | 0.841 | 0.757–0.935 | 0.001 |

*All landmark models adjusted for age, BMI, eGFR, smoking, and serum albumin. The sex × ΔT_LS−TH interaction remained statistically significant in the landmark analysis (p=0.001).*
